# T4 Integration of PT/OT in Burn Wound Care Increases Therapist Productivity

**DOI:** 10.1093/jbcr/irad045.004

**Published:** 2023-05-15

**Authors:** Amy Schwartzman, Blaire Balstad, Arek J Wiktor, Scott W Mueller

**Affiliations:** University of Colorado Burn and Frostbite Center, Aurora, Colorado; University of Colorado Anschutz, Denver, Colorado; University of Colorado, Aurora, Colorado; University of Colorado Burn and Frostbite Center, Aurora, Colorado; University of Colorado Burn and Frostbite Center, Aurora, Colorado; University of Colorado Anschutz, Denver, Colorado; University of Colorado, Aurora, Colorado; University of Colorado Burn and Frostbite Center, Aurora, Colorado; University of Colorado Burn and Frostbite Center, Aurora, Colorado; University of Colorado Anschutz, Denver, Colorado; University of Colorado, Aurora, Colorado; University of Colorado Burn and Frostbite Center, Aurora, Colorado; University of Colorado Burn and Frostbite Center, Aurora, Colorado; University of Colorado Anschutz, Denver, Colorado; University of Colorado, Aurora, Colorado; University of Colorado Burn and Frostbite Center, Aurora, Colorado

## Abstract

**Introduction:**

Inpatient physical (PT) and occupational (OT) burn therapists must balance complex patient factors such as daily wound care when attempting skilled therapy sessions, while also attaining specific productivity standards. These factors often lead to inefficient work flow and loss of productivity. The purpose of this study was to assess the productivity and feasibility of therapist integration into daily burn wound care.

**Methods:**

A quality improvement project was initiated by the burn therapy team at our ABA verified burn center. One full time equivalent (FTE) PT or OT was assigned to the burn wound care team five days a week, involving 6 therapists (3 PTs and 3 OTs). General duties included wound assessment, functional wound dressings, and skilled therapeutic interventions. The primary outcome measure was individual therapist productivity tracked 3 months pre and 3 months post project implementation. Productivity was calculated by taking total billable patient time divided by total workday time, with a goal of 50% productivity. Billed time units were also compared to goal units. The results were analyzed using a paired t-test to measure significance and averaged. The secondary outcome measure was program feasibility, assessed by a qualitative questionnaire taken by burn staff pre and post.

**Results:**

Overall therapist productivity increased, 49% pre implementation vs 54% post (p=0.0041). The difference of billed vs goal units increased, 5 pre vs 15.9 post (p= 0.00142). Proportion of therapists meeting target productivity increased, 57.1% pre vs 90.5% post (p=0.0052). Prior to program implementation, survey results found that 77% of burn staff (n=23 respondents) were favorable of project implementation, whereas 23% were either hesitant or indifferent. After 3 months post implementation, 95% of burn staff were favorable of therapist participation, 95% indicated ease of staffing burden, and 100% reported improved multidisciplinary communication. Furthermore, 100% of burn staff felt that the unit’s workflow had improved.

**Conclusions:**

Full time therapy participation in wound care increases therapist productivity and increases the likelihood of meeting target productivity in the future. The majority of burn staff actively supported this pilot program and felt that it eased staffing demands, improved unit workflow, and improved multidisciplinary communication. Future efforts should focus on measuring specific patient outcomes and cost as a result of therapist participation in daily wound care practice.

**Applicability of Research to Practice:**

All burn units should consider implementing a skilled burn therapist in wound care practice.

